# Real-World Outcomes Using a Spinal Cord Stimulation Device Capable of Combination Therapy for Chronic Pain: A European, Multicenter Experience

**DOI:** 10.3390/jcm10184085

**Published:** 2021-09-10

**Authors:** Jan Willem Kallewaard, Jose Francisco Paz-Solis, Pasquale De Negri, Maria Angeles Canós-Verdecho, Hayat Belaid, Simon J. Thomson, David Abejón, Jan Vesper, Vivek Mehta, Philippe Rigoard, Paolo Maino, Sarah Love-Jones, Isaac F. Peña, Simon Bayerl, Christophe Perruchoud, Renaud Bougeard, Cleo Mertz, Yu Pei, Roshini Jain

**Affiliations:** 1 Department of Anesthesiology and Pain Medicine, Rijnstate Hospital, 6815 Arnhem, The Netherlands; 2 Department of Neurosurgery, University Hospital La Paz, 28046 Madrid, Spain; pazsolis@icloud.com; 3 AORN S. Anna & S. Sebastiano, 81100 Caserta, Italy; pasquale.denegri@aslnapoli2nord.it; 4 Multidisciplinary Unit for Pain Treatment, University and Polytechnic Hospital La Fe, 46026 Valencia, Spain; angelescanos@hotmail.com; 5 Fondation Adolphe de Rothschild, 75019 Paris, France; hbelaid@for.paris; 6 Pain Management, Mid and South Essex University Hospitals NHSFT, Basildon SS16 5NL, UK; simon.thomson1@gmail.com; 7 Pain Unit, Hospital Universitario Quirónsalud, 28223 Madrid, Spain; dabejongonzalez@gmail.com; 8 Department of Functional Neurosurgery and Stereotaxy, University Medical Center Heinrich Heine University, 40225 Düsseldorf, Germany; jan.vesper@uniklinik-duesseldorf.de; 9 Pain & Anaesthesia Research Centre, St. Bartholomew’s Hospital, Bart’s Health, London EC1A 7BE, UK; vivek.mehta@mac.com; 10 Prismatics Lab & Spine Surgery and Neuromodulation Department, Poitiers University Hospital, 86021 Poitiers, France; Philippe.RIGOARD@chu-poitiers.fr; 11 Pain Management Center, Neurocenter of Southern Switzerland, 6900 Lugano, Switzerland; paolo.maino@eoc.ch; 12 Anaesthetics and Pain Management, Southmead Hospital, Bristol FCW5 P9, UK; Sarah.Love-Jones@nbt.nhs.uk; 13 Pain Unit, Virgen Del Rocio University Hospital, 41013 Sevilla, Spain; isaac.pena.sspa@juntadeandalucia.es; 14 Neurosurgery, Charité—Universitätsmedizin Berlin, 13353 Berlin, Germany; simon.bayerl@charite.de; 15 Pain Clinic, La Tour Hospital, 1217 Meyrin, Switzerland; christophe.perruchoud@latour.ch; 16 Clinique du Val d’Ouest, 69130 Écully, France; dr.bougeard@renaudbougeard.fr; 17 Boston Scientific Neuromodulation, 1861 Brussels, Belgium; cleo.mertz@bsci.com; 18 Boston Scientific Neuromodulation, Valencia, CA 91355, USA; yu.pei@bsci.com (Y.P.); roshini.jain@bsci.com (R.J.)

**Keywords:** chronic pain, combination therapy, customized stimulation field targeting, spinal cord stimulation, SCS

## Abstract

Given the differing mechanisms thought to underlie therapeutic sub- and supra-perception-based neurostimulative modalities, Spinal Cord Stimulation (SCS) systems designed for combined delivery of these approaches may help improve analgesic outcomes and quality of life, and reduce treatment failures. This multicenter, observational case-series evaluated 188 patients with chronic back and/or leg pain implanted with an SCS device capable of sequential or simultaneous delivery of sub-perception and supra-perception stimulation programming (i.e., combination therapy) at 16 in Europe. Following implantation, patients were provided with an array of advanced supra-perception programs (e.g., paresthesia-based SCS using multiple independent current sources), and a custom set of sub-perception programs optimized with specific waveforms and/or field shapes. A mean overall pain score of 7.9 ± 1.7 (Standard Deviation (SD)) was reported pre-trial (Baseline). Overall pain was reduced by 4.4 ± 2.8 points (NRS) at 3-months (*n* = 117) and at 12 months post-implant (*n* = 90), respectively (*p* < 0.0001). Substantial quality-of-life (EQ-5D-5L) improvement as assessed at last follow-up was also observed (*n* = 60). These results suggest that an implanted SCS device capable of combination therapy, while also enabled with patient-specific waveform optimization and stimulation field targeting capabilities, can enable highly effective pain relief and improve quality of life in patients suffering with chronic pain.

## 1. Introduction

Over the past decade, several new Spinal Cord Stimulation (SCS) approaches have been deployed in the clinical setting, including but not limited to high frequency (1–10 kHz), burst, and high density stimulation in an effort to improve patient outcomes using SCS to treat chronic pain [1,2,3,4]. While each of these innovations has in part contributed to the advancement of SCS as a therapeutic modality for chronic pain, patients are only typically provided with availability to these newer approaches via separate neuromodulation devices marketed by various device manufacturers. This fact alone has helped spur calls for the creation of more flexible systems capable of selectable and multi-programmable outputs that allow for customization of therapy [5,6,7]. However, it has also been suggested, on the basis of several studies that reported a sizable proportion of SCS-device revisions and/or explants occurred due to loss of therapeutic effectiveness, that devices with such capability may potentially be able to reduce or eliminate neural tolerance and/or loss of analgesic response over time in a substantial percentage of patients [6,7,8,9,10]. As such, a single SCS device providing multiple therapeutic modalities and highly adjustable programming options may help to better facilitate, identify, and personalize therapy that specifically works best for each patient over the course of treatment for chronic pain, and in selected cases, possibly avert the need for surgical revision or device explantation.

Numerous basic and clinical studies have now demonstrated that SCS can be therapeutically applied below the threshold of perception (i.e., sub-perception stimulation), in contrast to conventional methodologies traditionally utilized at or above perception threshold (i.e., supra-perception stimulation) [1,11,12,13,14]. Although distinct mechanisms that underlie both of these SCS approaches have not been definitively identified, an assortment of reported studies and clinical observations seem to suggest that differences likely do exist with regard to how the analgesic outcomes induced by sub- and supra-perception SCS are manifested [15,16,17,18]. Given this probability and the dynamic nature of the chronic pain experience, there is now a growing interest in the clinical application of sub- and supra-perception SCS as a combination therapy versus the exclusive use of either sub- or supra-perception SCS alone [6,19,20]. The commonly observed variable experience of chronic pain from patient-to-patient is thought to derive, at least in part, from the differing mechanisms that underlie the array of chronic pain syndromes and symptom complaints diagnosed in and imparted by SCS-implanted patients [21,22,23]. Thus, combining sub- and supra-modalities as a part of a continuous analgesic treatment regimen could provide an opportunity to better address clinical variability (i.e., intensity of pain, type of pain, pain location, pain etiology, other) that is known to occur within individual chronic-pain patients over time (intra-patient) and between separate individuals across this specific patient population (inter-patient).

To date, only a very limited number of peer-reviewed clinical studies have published outcomes in patients who have been implanted with devices designed to integrate sub- and supra-perception modalities. However, as these types of devices offering this capability become increasingly available to patients, we surmise that it will be important to monitor and track how these systems are used to treat pain as well as associated clinical outcomes [19,24,25,26]. To that end, we chose to embark on a multicenter, consecutive, observational, case-series consisting of a cohort of patients who used an SCS system engineered to provide patients with multiple waveform type and/or field shape programming options that also can simultaneously or sequentially deliver different neurostimulative modalities (i.e., sub- and supra-perception approaches) in combination. Here, we report the real-world outcomes with the use of the device per standard of care in those evaluated patients implanted with such a device across 16 centers in Europe.

## 2. Materials and Methods

### 2.1. Study Design

This is a multicenter, observational, retrospective case-series of permanently implanted patients who used an SCS system (Spectra WaveWriter, Boston Scientific, Marlborough, MA, USA) to treat chronic pain at 16 centers in Europe (Clinicaltrials.gov identifier: NCT01550575). Inclusion criteria required patients to have been previously treated with or eligible to receive a Spectra WaveWriter SCS system (Boston Scientific) and be 18 years of age or older. There were no exclusion criteria (apart from standard exclusion criteria for clinical use of a neuromodulatory device), per study protocol.

### 2.2. Study Setting and Participants

All study data were derived from 188 consecutively enrolled patients from 16 implanting centers in Europe. Ethics Committee approval was obtained from each site, and the study was conducted in accordance with GCP (ISO14155) guidelines and the Declaration of Helsinki.

### 2.3. Data Collection

All patients were pre-screened during the Trial Period where, per standard of care, patients attempted to use SCS to determine if they may be good candidates (prior to permanent implant). However, only those patients who received a permanent implant were included for data collection. Stimulation parameters including waveform and electric field shapes were adjusted according to patient preferences per standard-of-care. Due to the retrospective design of this study, data were collected from only those patients who had completed follow-up at the time of the data snapshot. As such, the number of patients assessed fluctuated across time, with a decreased number evaluated at later timepoints because data could not be attained from those who had not yet reached succeeding follow-up visits. To lessen potential partiality, only clinical site staff without sponsor involvement performed data collection from patients directly. Collection of demographic information, pain location, surgical history, and medical history was conducted. Numeric Rating Scale (NRS) scores and Percent Pain Relief (PPR) were collected as part of the chart review. Health-related quality-of-life assessment was conducted using the EuroQol Validity in Assessing the Quality of Life (EQ-5D-5L) standardized measure and was collected at patient follow-up visits [26]. Mean, median, and standard deviations were calculated for demographic data, NRS, and EQ-5D-5L scores. Patients reported their preference among multiple programming modalities, which was collected in their charts.

### 2.4. Statistics

For demographic data and NRS scores, means and standard deviations were determined. Score distribution was calculated for NRS and Percent Pain Relief. A paired *t* test with two-sided 0.05 significance level was used to calculate whether the mean reduction in baseline pain was greater than 0. A Kolmogorov–Smirnov Test was performed to confirm the normality of the change of NRS score.

### 2.5. Device Description

As described in detail in previous work [19], the SCS system utilized by patients included in this study (Spectra WaveWriter, Boston Scientific, Marlborough, MA, USA) uses anatomically guided neural targeting to deliver paresthesia-based SCS therapy using a three-dimensional finite element model of the spinal cord as well as an algorithm that has been designed to determine the exact amount of current needed at each relevant contact of interest, per a user-specified central point of stimulation on the dorsal column [27]. Patients included in this study were implanted with either an 8-contact percutaneous lead with 1, 4, or 6 mm edge-to-edge spacing and 31, 62, or 66 mm span (Linear or Linear ST, Boston Scientific, Marlborough, MA, USA) or a 16-contact percutaneous lead (Infinion CX, Boston Scientific, Marlborough, MA, USA) with 1 mm edge-to-edge spacing and 67 mm span. In order to effectively identify the best “sweet spot” for sub-perception SCS, the device can generate a comprehensive electrical field along a rostrocaudal orientation to enhance the probability of engaging correspondingly associated inhibitory interneurons. Additionally, as described prior, clinical application of higher frequency (i.e., up to 1200 Hz) sub-perception SCS and lower frequency supra-perception (paresthesia-based) SCS as a combination therapy can be accomplished by the SCS system used by patients in this study such that multiple SCS waveforms can be operated in the same program (simultaneous delivery) [19]. Moreover, specifically directing electrical fields to various neural targets can be accomplished using available tightly spaced lead contacts and multiple independent current controls as well as an available sub-perception-based electric-field-targeting algorithm (Contour, Boston Scientific). This algorithm has been engineered to allow for highly manipulatable, conformational fine-tuning of SCS fields that in turn can provide for enhanced individual customization of therapy (i.e., when compared to older generation SCS systems). Further, the SCS system used by patients described in this report also can be operated using different stimulation settings (e.g., amplitude, pulse-width, and frequency) that in accordance with known parameter relationships can be modified to alter the total amount of charge delivered to neural tissue per unit time (i.e., neural dosing) in an automated or sequentially scheduled manner (i.e., waveform automation), as described previously [28]. This capability also provides for multiple available sub- or supra-perception-based algorithmic programming options (e.g., standard rate, burst (active recharge), microburst, high rate, custom stimulation field configuration) that can be automatically evaluated and sequenced during the programming optimization process without manual intervention by the physician, programmer, and/or patient (i.e., patient being remote). This feature is thought to be especially beneficial given the previously reported latency period of analgesic onset when using conventional sub-perception waveforms (typically a 1–3 day until “wash-in” or duration until maximum pain relief) [29,30,31].

## 3. Results

**Figure 1:**
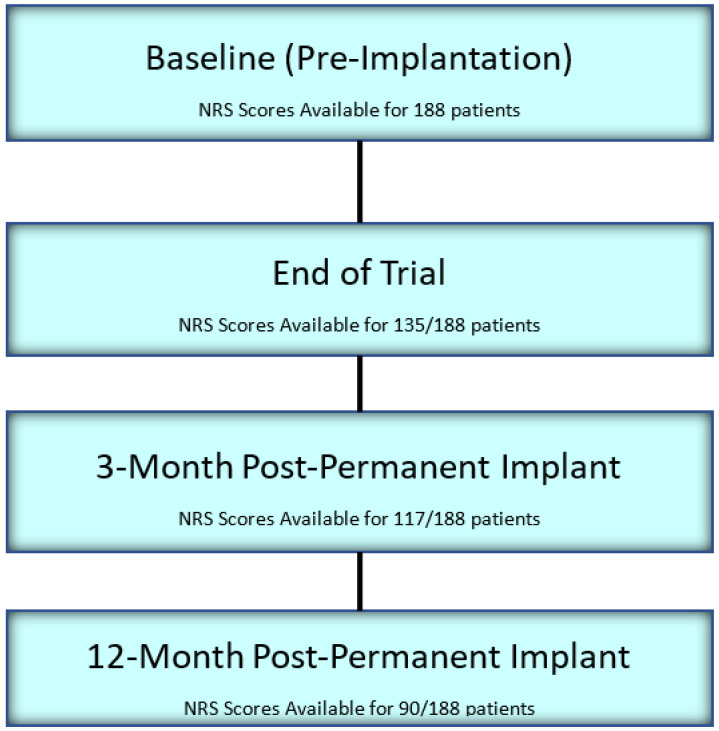
Patient Disposition.

In this study, a total of 188 permanently implanted patients were evaluated. Data were collected and assessed from 135, 117, and 90 patients at the completion of trial implantation, 3 months after permanent implant, and 12 months after permanent implant, respectively (Figure 1). Among all included patients, 101 of them were female. On average, patients were 60.0 ± 12.3 years of age with 85.6% reporting low back and leg pain, and were diagnosed with one or more of the following: Lumbosacral Radiculopathy (21%), Failed Back Surgery Syndrome (64%), and Compressive Myelopathy from Spinal Stenosis (9%). Baseline (pre-trial) NRS scores were available for all patients, with a mean of 7.9 ± 1.7 (*n* = 188). The mean follow-up (last follow up) duration was found to be 296 ± 207 days (*n* = 187) (Table 1).

**Figure 2:**
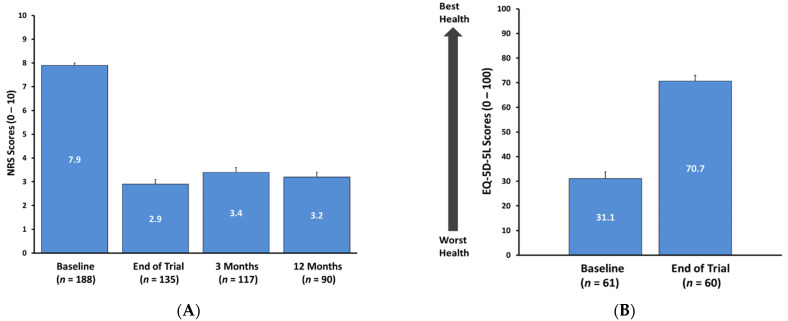
(**A**) Baseline, end of trial, and 3 and 12 months post-implant mean overall NRS pain scores. Error bars signify standard error (SE). *p* < 0.0001. (**B**) Overall quality of life, as assessed by EQ-5D-5L at Baseline and Last follow up. Error bars represent standard error (SE).

In patients assessed at the end of trial and 3 and 12 months following implantation, mean NRS score was reduced to 2.9 ± 2.3 (*n* = 135), 3.4 ± 2.2 (*n* = 117), and 3.2 ± 2.3 (*n* = 90), respectively (*p* < 0.0001) (Figure 2A). Some patients had not reached these specified timepoints, thus resulting in variable sample size across follow-up visits. At these timepoints, this degree of pain reduction correlated with a 68.4% (3 months) and 70.0% (12 months) responder rate, as delineated by the percentage of those with a ≥50% improvement in overall pain versus Baseline using NRS scores. Per the range of mean NRS pain scores acquired from all 188 assessed patients at last follow-up, 53% (100/188) reported overall pain NRS scores of 3 or less (data not shown). A similar trend was noted among low back pain scores. Among those patients who reported leg pain, a mean NRS pain score of 4.7 ± 3.0 (7.6 → 2.8, *n* = 96) was noted at 3 months, and this was maintained up to 12 months’ follow-up (7.6 → 2.9, *n* = 78). 

Quality of life improvement was noted based on EQ-5D-5L assessment. Among those included patients for whom assessment was carried out, a clinically meaningful mean improvement was noted as reflected by a 42-point increase in EQ-5D-5L score (Figure 2B).

**Figure 3:**
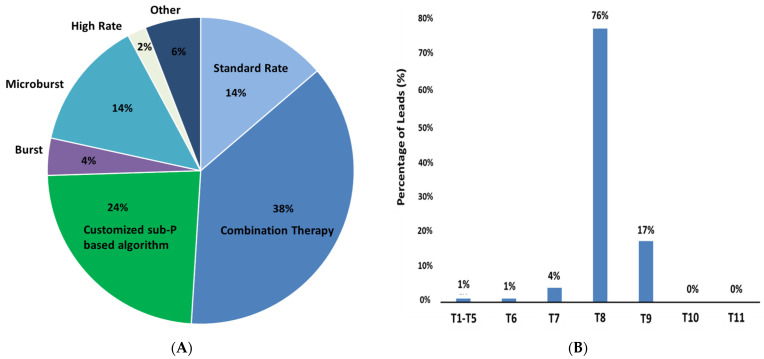
(**A**) Patient preferred programs at last follow-up. Combination Therapy is the ability to allow for simultaneous delivery of sub- and supra-perception stimulation modalities. Customized sub-perception-based algorithm designed to engage anti-nociceptive terminals over a broader area producing a stronger dorsal horn effect. (**B**) Vertebral location of top of implanted dual 16-contact leads (*n* = 69).

Thirty-eight percent of patients preferred programs utilizing combination therapy (defined as simultaneous or sequential delivery of sub- and supra-perception stimulation) among the multiple neurostimulative modalities available to patients in this study (Figure 3A). Other SCS programming approaches that were preferred by patients in this cohort included use of a customized, sub-perception-based field shape algorithm (24%), standard rate (14%), and microburst stimulation (14%). Patients may have preferred multiple programs.

Of the 188 evaluated patients, 69 patients (mean age = 55.8 ± 11.3 years, 38 female) were implanted with dual 16-contact percutaneous leads with 1 mm edge-to-edge spacing and 67 mm span. Seventy-six percent of leads placed were found to span multiple vertebral levels (electrode tip starting at T8) as shown in Figure 3B. In this particular sub-group of patients, a 4.5-point improvement (*n* = 53, *p* < 0.0001) was reported at 3 months that was sustained up to 12 months (4.6-point improvement, *n* = 46, *p* < 0.0001). At last follow-up (mean duration of 382 days), a 4.3-point improvement was noted (7.5 → 3.2, *n* = 69). A significant improvement in quality of life was also noted as assessed by EQ-5D-5L (Baseline: 18.6, *n* = 28; Last follow up: 74.1, *n* = 29).

## 4. Discussion

The outcomes of this international, real world, observational study demonstrate that patients with chronic pain who used an SCS system capable of combining sub- and supra-perception stimulation modalities as part of an available array of waveform and stimulation field targeting algorithms can achieve successful clinical outcomes that are sustained out to 1-year follow-up. These data are comparable with those reported in previous studies that assessed patients implanted with devices capable of providing options for various neurostimulative programming modalities [19,30,31,32,33,34]. In so doing, this study adds to the compendium of mounting evidence suggesting that patients do appear to benefit when they are able to selectively choose among an assortment of neurostimulation paradigms, contingent on their specific needs at any given time, during the course of their SCS therapy. Importantly, in this study, patients had access to device technologies capable of combining sub- and supra-perception stimulation in a simultaneous or sequential manner (combination therapy) as well as newly available lead types with features thought to be more advantageous versus other traditional lead designs (e.g., increased lead span allowing for coverage over multiple vertebral levels). While combination therapy was not used by all those evaluated in this cohort, this programming approach was found to be the most preferred, followed secondarily by preference for use of a contoured field shape algorithm enabling customized, sub-perception-based stimulation field targeting. The study did not collect information on why patients preferred these programs. However, it may be speculated that using SCS-based methodologies designed to be applied in a patient-specific manner may offer the advantage of providing markedly improved analgesia resulting from the enhanced ability to optimize or “tailor” treatment to the individual as well as to more readily modify therapy in response to specific changes in the experience of pain that can often occur within and across patients. Furthermore, the option to choose multiple programs may also help prevent loss of efficacy (or habituation) over time.

Sub- and supra-perception SCS modalities are thought to elicit pain relief through distinct mechanisms, but to date there still remains no concrete understanding of how either of these approaches induces analgesia, although several theories have been proposed [35,36,37]. However, one commonality among the various proposed models is that of the importance of dorsal horn targeting. The dorsal horn of the spinal cord is known to consist of neurons that play a direct role in the processing of sensory information delivered to various regions of the brain, and several pre-clinical animal studies now strongly suggest that neuronal elements in the spinal dorsal horn play a critical role in facilitating transmission of pain signals [38,39,40,41,42,43]. Interestingly, engagement with synaptic terminals of rostrocaudally oriented inhibitory interneurons in the superficial dorsal horn is thought to occur when the applied neural dose has been effectively optimized [28,44,45]. In addition, these neuronal terminal sites may be more favorably polarized over axons of passage or cell bodies when using specifically engineered near-field electrical stimulation geometries that maximize the local electrical field [46,47,48,49,50]. It is on the basis of these and other related studies that the SCS device under evaluation in this report was accordingly rationally designed, so as to allow for selective modulation of precise neuronal targets that occur in the dorsal horn (as opposed to dorsal column elements) using a novel, sub-perception-based stimulation field targeting algorithm that can be highly customized to the individual patient. Moreover, this algorithm can be simultaneously or sequentially combined with conventional supra-perception (paresthesia-based) stimulation programming to produce a combination therapy as was available to patients in this study. Though not all patients in this cohort preferred use of combination therapy or use of the customized field targeting algorithm alone, we nonetheless surmise that SCS devices offering a high degree of flexibility to patients are likely well-suited to help address concerns regarding patient-to-patient variability and treatment complications that may lead to surgical revision and/or device explantation, at least in part, because of the presumed ability to engage different putative mechanisms of action that are thought to govern both sub- and supra-perception stimulation. Thus, we postulate that with increased understanding of the underlying neural circuitry involved in the mechanisms that mediate SCS-induced analgesia, devices with these and other rationally designed technological capabilities may likely play an important role in helping to further improve the clinical outcomes associated with the use of SCS for management of chronic pain as well as make a significant contribution toward the broader, overarching pursuit of deploying therapeutic medical devices that foster the on-going advancement toward the practical, real-world delivery of more personalized medicine.

Particular limitations of this study include the retrospective nature of the data collection, thereby restricting this analysis to only the available clinical data contained in medical charts of evaluated patients, as collected per standard of care. As such, functional outcomes such as assessment of disability and opioid drug medication reduction were not able to be obtained. Moreover, this study does not allow for the evaluation of outcomes associated with the specific modes of SCS treatment that patients were able to utilize. Future studies will be required to evaluate whether meaningful associations exist among those who use particular waveforms and when and which clinical correlates, if any, may be associated with specific stimulation programming paradigms. Additionally, whether the utilization of combination therapy, as an available feature on an SCS device that allows for patient-selective waveform programming options and custom targeting of neurostimulation fields, actually can help foster a reduction in the real-world incidence of surgical revision or device explant is also an important question for future clinical investigations to now consider. Further, results obtained in this study also indicate that placements of newly available 16-contact percutaneous leads designed to span multiple vertebral levels, as used by a sub-set included for analysis in this study, are overwhelming performed in a similar manner across patients and are associated with positive clinical outcomes. These preliminary data thus can serve as a basis for future investigation of patients using these newer lead types, when also implanted with SCS devices equipped with enhanced neurostimulative targeting capabilities.

## 5. Conclusions

This multicenter, international, real-world, observational study demonstrates that a programmable SCS device engineered to integrate different sub- and supra-perception modalities as a combination therapy, while also providing for selectable waveform options and patient-specific stimulation field targeting, can allow for highly effective pain relief and improved quality of life. An implantable device fully equipped with these capabilities may be a valuable tool to personalize SCS therapy and to reduce monotherapeutic treatment failures that provoke surgical revision and/or device explantation. Thus, providing multiple neurostimulative programming options offers substantial autonomy and versatility to patients over the total course of their experience using an SCS-based therapeutic strategy for treatment of chronic pain. Further studies with long-term follow-up are needed to explore the longevity and impact of the use of multiple programs and patient preferences.

## Figures and Tables

**Table 1:** Baseline and demographic characteristics in analyzed patients (*n* = 188).

Gender—Females (%)	53.1% (101/188)
Age (Mean (SD))	60.0 (12.3) years *n* = 180
Key Diagnosis (patients may have multiple diagnoses)	Lumbosacral Radiculopathy 21%
	Failed Back Surgery Syndrome 64%
Pain Location (%)	Low Back and Legs (85.6%)
Baseline NRS (Mean (SD))	7.9 (1.7) *n* = 188
Follow-up duration (Mean (SD))	296 (207) days *n* = 187

## Data Availability

The data, analytic methods, and study materials for this clinical study Data Availability Statement will be made available to other researchers in accordance with Boston Scientific Data Sharing Policy (https://www.bostonscientific.com, accessed on 6 September 2021).
